# Dynamics of two-process astrocyte networks

**DOI:** 10.1186/1471-2202-14-S1-P433

**Published:** 2013-07-08

**Authors:** Daniel Maruyama, Michal Zochowski

**Affiliations:** 1 Physics, Univ. of Michigan, Ann Arbor, MI 48109, USA; 2 Biophysics, Univ. of Michigan, Ann Arbor, MI 48109, USA

## 

Computation in the brain is often thought of as being carried out solely by neurons without regards to their supporting cells. Recent work suggests that astrocytes may play a role alongside neurons in information processing. We investigated what types of spatio-temporal patterning can be supported for such a network. Namely the astrocyte networks contained both fast speed direct connections, representing gap junction coupling, and slower speed extracellular connections, representing local ATP diffusion or changes in local potassium concentration. We investigated what spatio-temporal dynamics results from the interplay between these two processes. We observed each process individually drive network activity and saw competition between the local and direct connections.

